# Association between insulin resistance trajectories and perinatal complications: a retrospective cohort study

**DOI:** 10.3389/fendo.2026.1746255

**Published:** 2026-03-30

**Authors:** Lisha Ye, Lixia Shen, Shaofeng Zhang, Caixia Zhu, Shiqin Cai, Jingwan Huang, Lepei Xie, Haitian Chen

**Affiliations:** Department of Obstetrics and Gynecology, Guangdong Provincial Clinical Research Center for Obstetrical and Gynecological Diseases, The First Affiliated Hospital of Sun Yat-sen University, Sun Yat-sen University, Guangzhou, China

**Keywords:** gestational diabetes mellitus, hypertensive disorders of pregnancy, insulin resistance, modeling, pregnancy outcomes

## Abstract

**Background:**

During pregnancy, the maternal body undergoes adaptive regulation to support the normal growth and development of the fetus, such as the occurrence of insulin resistance (IR). When the degree of IR progresses continuously and is significantly higher than the normal range in the corresponding period, it will increase the risk of perinatal complications, including gestational diabetes mellitus (GDM) and hypertensive disorders of pregnancy (HDP). The study is to investigate the correlation and influenced factors between different levels of IR during pregnancy and adverse pregnancy outcomes, and to suggest the critical threshold for predicting pathological IR.

**Method:**

By applying the Group-based Trajectory Model (GBTM), the trajectories’ classification based on IR index changes was constructed, including fasting serum insulin (FINS), insulin resistance homeostasis model assessment (HOMA-IR), and the quantitative insulin sensitivity check index (QUICKI), to analyze the differences in the differentiation of IR evaluation indicators. We used the Poisson Regression Model to analyze the correlation between various IR changing trajectories and adverse pregnancy outcomes.

**Results:**

Four gradient differences in IR change trajectories were fitted in the population using three IR evaluation indicators respectively, including low level, moderate level, high level and low-high level. For FINS trajectories, the GDM risk of the moderate level trajectory (aRR 1.73, 95% CI 1.21-2.47) and the high-level trajectory (aRR 2.40, 95% CI 1.58-3.65) showed a gradient increase. The low-high level trajectory, which only sharply increased in late pregnancy, exhibited a significantly higher difference in HDP risk (aRR 6.45, 95% CI 1.78-23.33) compared to the high level (aRR 5.86, 95% CI 2.41-14.25). In this study, the early pregnancy IR value of the population was used to predict GDM exhibited similar predictive ability (AUC 0.607, P<0.001). For predicting HDP, FINS showed the highest AUC value of 0.740 (P<0.001), comparing to HOMA-IR and QUICKI showed slightly lower AUC values of 0.73.

**Conclusion:**

There are various subtypes of IR trajectories in pregnant women, which are significantly correlated with adverse pregnancy outcomes.

## 1 Introduction

Insulin resistance (IR) is a common physiological phenomenon observed in pregnant women, particularly after the mid-trimester. This is an essential role to process elevated level glycolipid metabolites in fetal development (1, 2). On the differentiation between pathological and physiological IR and the insulin secretory response, there is still no unequivocal criteria and consensus (3).

Pathological IR during pregnancy is linked to adverse perinatal complications, including gestational diabetes mellitus (GDM) (4), hypertensive disorders of pregnancy (HDP) (5), cesarean delivery, macrosomia, shoulder dystocia, neonatal hypoglycemia, a higher long-term risk of developing diabetes and cardiovascular disease. The incidence of perinatal complications decreased in clinical trials aimed at improving IR (6, 7), suggesting that monitoring the fluctuations of IR may contribute to avoid adverse perinatal outcomes. Nevertheless, research about the associations between perinatal complications and IR throughout pregnancy is still lacking, it is imperative to conduct some studies on IR relevant trajectories.

Group-based trajectory modeling (GBTM) is a statistical technique used to identify distinct subgroups of individuals who follow similar progressions of an outcome over time (8). Employing trajectory groups as a statistical tool allows for the identification of pregnancy populations that share similar developmental patterns; it can also be understood as a process of longitudinal clustering (9). This method is well-established in the analysis of longitudinal data in developmental criminology, sociology, and psychology, and has been extensively applied in obstetric research, including analyses of glycemic trajectories (10) and intergenerational behavioral trajectories (11).

This study aims to analyze the potential heterogeneous trend of changes in gestational IR levels by GBTM, identify the critical threshold for risk stratification of pathological IR, and explore the correlation and influencing factors between varying levels of IR in adverse pregnancy outcomes.

## 2 Methods

### 2.1 Study design

Pregnant women who received prenatal care and delivered at The First Affiliated Hospital of Sun Yat-sen University (FAH-SYSU) between June 1, 2021, and August 31, 2023, were included in this study. Three trimesters of pregnancy (9-14, 24-28, and 35–40 gestational weeks) should be covered by the routine antenatal examination on fasting serum insulin (FINS) and fasting plasma glucose (FPG) levels.

Inclusion criteria of the study included (**Supplementary Figure S1**): 1) singleton pregnancy; 2) Maternal age ≥ 18 years old; 3) delivery gestational age ≥ 28 weeks; 4) The initial prenatal examination time is less than 14 weeks.

Exclusion criteria were as follows: 1) pregestational diabetes mellitus (PGDM) including preexisting type 1 or type 2 diabetes mellitus and overt diabetes diagnosed during pregnancy, 2) multiple pregnancies, 3) use of medications that affect glucose levels (metformin, steroid hormone, atosiban, and insulin therapy), 4) individuals with incomplete data.

### 2.2 Study sample assessment

The routine prenatal visits provided all the necessary basic information and assessments of laboratory examinations. Participants visited at <14 weeks’ gestation (early pregnancy), 24–28 weeks’ gestation (mid pregnancy), and 35–40 weeks’ gestation (late pregnancy). At the first antenatal visit, they had basic information collection regarding age, personal and family history, gestation history, race and native area, employment, education, marital status, and fertilization method. Weight and height were also measured at the initial information uploading and follow-up visits. During each scheduled visit, blood samples were obtained from participants after an overnight fast lasting 8–10 hours. These samples were collected using drying vacuum tubes and subsequently analyzed for HbA1c (early and mid), glucose, and insulin levels.

Participants in the mid-pregnancy visit underwent a 75-g oral glucose tolerance test (OGTT); blood samples were taken separately in fasting, 60 and 120 minutes after the glucose load, then simultaneously analyzed for levels of plasma glucose and serum insulin. Blood sample results were reported to obstetricians and dietitians for consultations, if necessary, to adjust diet as well as lifestyle and reach the targeted glycemic control range. All processes were done in the central laboratory of FAH-SYSU.

### 2.3 Perinatal complications

Data on the following perinatal outcomes were collected from the electronic medical record system: gestational diabetes mellitus diagnosed according to the 75g-OGTT tests in 24–28 weeks (12), cesarean delivery, hypertensive disorders of pregnancy (including gestational hypertension, pre-eclampsia, and eclampsia, according to Chinese criteria) (13), dystocia, postpartum hemorrhage (within 24 hours after delivery, vaginal delivery≥500ml; cesarean section≥1000ml), neonatal admission to neonatal intensive care (NICU), and gestational age specific birthweight categories based on a 2019 Chinese reference population (small-for-gestational age [SGA],<10th percentile; and large-for--gestational age [LGA], >90th percentile]) (14).

### 2.4 Individual-level multidomain factors

Data on individual-level factors across two domains were obtained: 1) sociodemographic characteristics, including age at delivery, ethnic group (Han or other minorities), residential area (Guangdong province or other provinces), multiparity, and pre-pregnancy body mass index (BMI, classification according to Chinese BMI Classification Reference Standards). 2) risk factors for hyperglycemia during pregnancy, including a history of GDM or macrosomia, first-degree relative history of diabetes, in a FPG levels of 5.1-6.9mmol/L in early pregnancy (15), and HbA1c 5.9%-6.4% (41 mmol/mol) in early pregnancy (16).

### 2.5 Insulin resistance assessment

IR relevant index was measured utilizing quantitative insulin sensitivity check index (QUICKI) (17) and homeostasis model assessment of insulin resistance (HOMA-IR) (18). They were calculated as follows:


HOMA−IR = (FPG in mmol/L ×FINS in pmol/L) / 22.5

QUICKI = 1 / [ Log (FPG in mg/dl) + Log (FINS in μU/ml)]

### 2.6 Statistical analysis

Baseline characteristics of the participants were presented as means and standard deviations (SD) for continuous variables, and as percentages for categorical variables. Unpaired t-tests were used to examine disparities in means for continuous variables, and *X*^2^ tests were used to evaluate the independence of categorical variables. The Poisson trend test was used to ascertain the significance, denoted as *P*. *P* values were corrected according to the Benjamini-Hochberg procedure controlling the false discovery rate, and less than 0.05 were considered significantly different. The analyses were conducted using IBM SPSS Statistics version 29.0. To account for the correlation of outcomes among multiple pregnancies from the same individual, we applied generalized estimating equations (GEEs) with robust (sandwich) standard errors.

Group-Based Trajectory Modeling (GBTM) was used to ascertain the trajectories of IR associated indicators within subgroups. The”traj”being plugged into STATA MP version 16 (Stata Corp) (8). We evaluated models fit for different subgroups and types of potential trajectories in order to identify patterns throughout pregnancy and possible differentiated subtypes within them. The number and shape (up to cubic) of trajectories were determined using censored normal models, with order selection based on the statistical significance of polynomial terms (P<0.05). The selection of the optimal model was based on multiple criteria: 1) the highest Bayesian information criterion, 2) an average posterior probability exceeding 0.7, 3) the inclusion of at least 1% of all cases in each subgroup, 4) and the ability to provide meaningful clinical interpretation. GBTM analysis was conducted on FINS, QUICKI, and HOMA-IR.

We performed multivariable Poisson regression models to estimate adjusted relative risks (aRR) and 95% confidence intervals (CI) to investigate the associations among gestational trajectories, perinatal complications, and individual-level multidomain variables (19). Sequential multivariable models were developed, comprising the unadjusted models (model 1), adjusted for sociodemographic characteristics (model 2), and sociodemographic characteristics plus risk factors for hyperglycemia during pregnancy (model 3). For the first part, IR trajectories were utilized as the primary exposure variable, while the perinatal outcomes were considered as the outcome of interest. Then the classification with the greatest clinical significance would be chosen and subjected to a complete analysis. For the second part, individual-level multidomain factors were utilized as the exposure, while the trajectories were considered as the outcome of interest, which aimed to explore the underlying risk factors that could modify the association between IR trajectories and perinatal complications.

## 3 Results

### 3.1 Characteristics of the study population

Among 5295 individuals who visited our center during the study period, 1504 individuals were finally included in our analysis. The baseline characteristics of the study were demonstrated in **Table 1**. With 378 participants (25.1%) were advanced age and 144 (9.5%) were defined as overweight and obesity, 583 (38.3%) came from the other areas beyond Guangdong, and 49 (3.3%) of ethnic minorities.

**Table 1:** Characteristics throughout pregnancy among 1504 women in different serum insulin level trajectories.

Factor^a^	F1^b^	F2^b^	F3^b^	F4^b^	Total	P-value^c^
Participants (n)	433( 28.8 )	826 (54.9 )	216 ( 14.4 )	29( 1.9 )	1504	
Age at delivery	31.77 ± 4.07	31.80 ± 4.18	31.88 ± 4.28	31.24 ± 4.04	31.80 ± 4.16	0.780
Advantaged maternal age (%)	111 ( 25.6 )	208 ( 25.2 )	54 ( 14.3 )	5 ( 17.2 )	378 ( 25.1 )	0.796
BMI(kg/m2)						
<18.5	115 ( 26.6 )	100 ( 12.1 )	4 ( 0.7 )	7 ( 24.1 )	226 ( 15.0 )	<0.001*
18.5-24.9	310 ( 71.6 )	667 ( 80.8 )	139 ( 64.4 )	18 ( 62.1 )	1134 ( 75.4 )	
25.0-29.9	8 ( 1.8 )	56 ( 6.8 )	65 ( 30.1 )	4 ( 13.8 )	133 ( 8.8 )	
≥30	0 ( 0.0 )	3 ( 0.4 )	8 ( 3.7 )	0 ( 0.0 )	11 ( 0.7 )	
Race						
Han	419 ( 96.8 )	800 ( 96.9 )	208 ( 96.3 )	28 ( 96.6 )	1455 ( 96.7 )	0.982
Ethnic minorities	14 ( 3.2 )	26 ( 3.1 )	8 ( 3.7 )	1 ( 3.4 )	49 ( 3.3 )	
Residential area						
Guangdong	261 (60.3 )	506 ( 61.3 )	101 ( 67.8 )	17 ( 58.6 )	921 ( 61.2 )	0.928
The other areas	172 (39.7 )	320 ( 38.7 )	79 ( 36.6 )	12 ( 41.4 )	583 ( 38.8 )	
Natural pregnancy	265 ( 61.2 )	535 ( 64.8 )	146 ( 67.6 )	15 ( 51.7 )	961 ( 63.9 )	0.378
Nulliparous	288 ( 66.5 )	535 ( 64.8 )	138 ( 63.9 )	18 ( 62.1 )	979 ( 65.1 )	0.928
Family history of diabetes	29 ( 6.7 )	73 ( 8.8 )	29 ( 13.4 )	5 ( 17.2 )	136 ( 9.0 )	0.080
Newborn gender	217 ( 50.1 )	429 ( 51.9 )	109 ( 50.5 )	14 ( 48.3 )	769 ( 51.1 )	0.982
GDM	35 ( 8.1 )	122 ( 14.8 )	48 ( 22.2 )	4 ( 13.8 )	209 ( 13.9 )	<0.001*
HDP	7 ( 1.6 )	29 ( 3.5 )	24 ( 11.1 )	3 ( 10.3 )	63 ( 4.2 )	<0.001*
Cesarean delivery	181 ( 41.8 )	364 ( 44.1 )	122 ( 56.5 )	11 ( 37.9 )	678 ( 45.1 )	0.015*
dystocia	39( 9.0 )	68 ( 8.2 )	13 ( 6.0 )	4 ( 13.8 )	124 ( 8.2 )	0.576
Preterm birth	2 ( 0.5 )	8 ( 1.0 )	1 ( 0.5 )	0 ( 0.0 )	11( 0.7 )	0.780
PPH	29 ( 6.7 )	72 ( 8.7 )	13 ( 6.0 )	4 ( 13.8 )	118 ( 7.8 )	0.423
Birth weight categories^d^						
LGA&macrosomia	20 ( 4.6 )	37 ( 4.5 )	18 ( 8.3 )	1 ( 3.4 )	76 ( 5.1 )	0.250
SGA	19 ( 4.4 )	39 ( 4.7 )	5 ( 2.3 )	1 ( 3.4 )	64 ( 4.3 )	0.576
NICU admission	30 ( 7.0 )	67 ( 8.1 )	15 ( 6.9 )	3 ( 10.3 )	115 ( 7.6 )	0.780
HbA1c						
Early	5.15 ± 0.30	5.17 ± 0.29	5.22 ± 0.32	5.20 ± 0.50	5.17 ± 0.30	0.030*
Middle	4.84 ± 0.28	4.93 ± 0.30	5.05 ± 0.34	4.80 ± 0.48	4.92 ± 0.31	<0.001*
Glucose						
Early	4.17 ± 0.36	4.27 ± 0.37	4.39 ± 0.37	4.22 ± 0.31	4.26 ± 0.37	<0.001*
Late	3.84 ± 0.38	4.00 ± 0.44	4.21 ± 0.64	6.18 ± 1.23	4.02 ± 0.58	<0.001*
Insulin						
Early	3.93 ± 1.46	6.53 ± 2.31	11.81 ± 4.06	5.88 ± 2.57	6.53 ± 3.47	<0.001*
Late	5.27 ± 1.83	9.21 ± 3.15	17.57 ± 7.08	71.61 ± 31.88	10.48 ± 10.98	
HOMA-IR						
Early	0.74 ± 0.31	1.25 ± 0.50	2.31 ± 0.85	1.11 ± 0.52	1.25 ± 0.72	<0.001*
Middle	0.86 ± 0.28	1.49 ± 0.48	2.74 ± 0.99	3.34 ± 1.68	1.48 ± 0.80	<0.001*
Late	0.91 ± 0.35	1.65 ± 0.65	3.34 ± 1.68	13.39 ± 2.44	1.90 ± 1.99	<0.001*
QUICKI						
Early	0.412 ± 0.309	0.375 ± 0.022	0.340 ± 0.017	0.386 ± 0.031	0.381 ± 0.033	<0.001*
Middle	0.398 ± 0.024	0.363 ± 0.019	0.332 ± 0.015	0.375 ± 0.027	0.375 ± 0.027	<0.001
Late	0.397 ± 0.029	0.359 ± 0.021	0.324 ± 0.018	0.260 ± 0.015	0.363 ± 0.036	<0.001
OGTT						
0h	4.07 ± 0.32	4.21 ± 0.34	4.37 ± 0.45	4.15 ± 0.32	4.19 ± 0.37	<0.001*
1h	7.28 ± 1.54	7.70 ± 1.60	8.16 ± 1.66	7.60 ± 1.45	7.64 ± 1.61	<0.001*
2h	6.30 ± 1.39	6.62 ± 1.34	7.02 ± 1.40	6.38 ± 1.48	6.58 ± 1.38	<0.001*

BMI, body mass index(calculated by pre-pregnancy weight in kilograms divided by height in meters squared); GDM, gestational diabetes mellitus; HDP, hypertensive disorders of pregnancy; PPH, postpartum hemorrhage; LGA, large for gestational age; SGA, small for gestational age; NICU, neonatal intensive care unit; HOMA-IR, homeostasis model assessment of insulin resistance; QUICKI, quantitative insulin sensitivity check index; OGTT, oral glucose tolerance test.

a Continuous variables were presented as mean (SD).

b F1,F2,F3,F4 refer to serum insulin level trajectory 1–4 modeled by GBTM.

c P-values were calculated by Kruskal–Wallis test for continuous variables and Chi-square test for categorical variables. *P<0.05: Adjusted for multiple testing using the Benjamini-Hochberg (BH) false discovery rate (FDR) correction to control for the family-wise error rate.

d SGA, LGA: Birth weight categories derived using sex and gestational age-specific percentiles calculated using a 2019 Chinses reference population.

The incidence of GDM, HDP, PPH, cesarean section, and dystocia were 13.9%, 4.2%,7.8% and 8.2% respectively. In addition, only 11 infants (0.7%) were born preterm in our study, which may be attributed to lacking measurement of fasting blood glucose and insulin in the late stage of pregnancy.

### 3.2 Gestational trajectories of IR index

**Figure 1:**
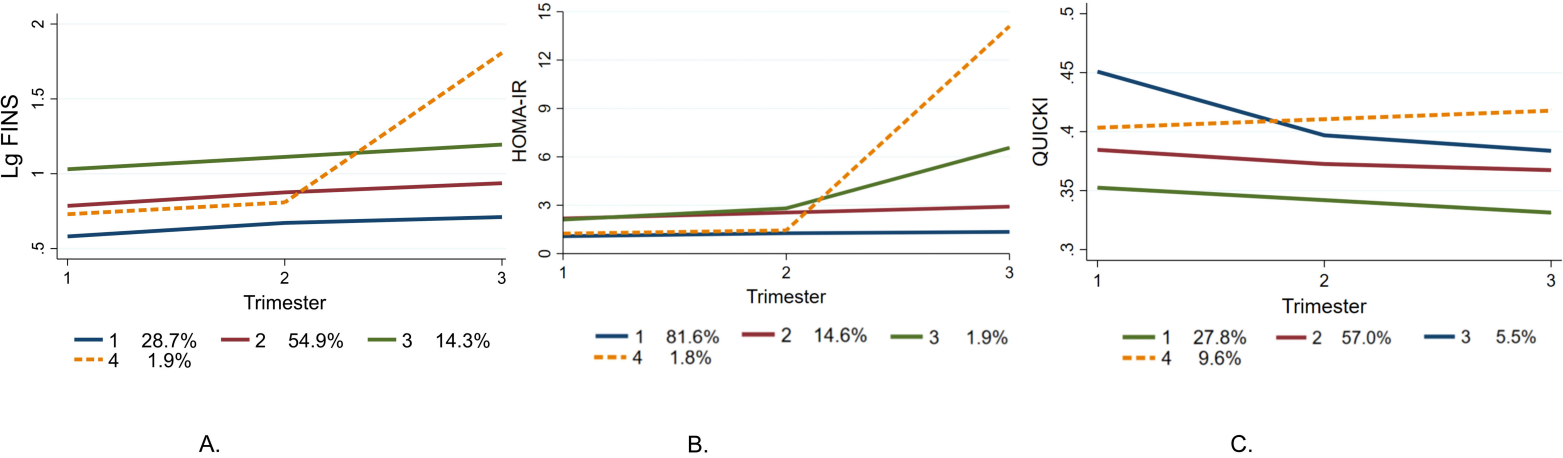
Best-fit trajectories during pregnancy. **(A)** FINS Trajectories, **(B)** HOMAR-IR Trajectories, **(C)** QUICKI Trajectories. FINS, fasting Insulin; QUICKI, quantitative insulin sensitivity check index; HOMA-IR, homeostasis model assessment of insulin resistance.

Finally, as shown in **Figure 1**, our model fitted four trajectories of insulin resistance indices respectively (**Supplementary Table S1**). The optimal polynomial order for each trajectory group was determined by progressively eliminating non-significant higher-order terms (starting from a cubic function) based on the significance of parameter estimates (P<0.05) in **Supplementary Table S2**.

In FINS model, each case is divided into one of four subgroups called Trajectory 1(F1), Trajectory 2(F2), Trajectory 3(F3), and Trajectory 4(F4), which stand for low, moderate, high, and low-high levels of insulin trajectory, respectively. Among them, there are 826 individuals in F2, accounting for 54.9%, with the closest FINS level to the population average. It could be observed that insulin secretion gradually increase as the gestational week progresses in a subset of participants. Specifically, F4 exhibits a unique insulin pattern during pregnancy,which is similar to F2 before 28 weeks but has a sharp elevation in late pregnancy to a level even higher than F3.

HOMA-IR had also been divided into four subgroups: low level Trajectory 1 (H1), moderate level Trajectory 2 (H2), high level Trajectory 3 (H3), and low-high level Trajectory 4 (H4). Among the four distinct curves representing the optimal selection of HOMA-IR, it was evident that the level of differentiation was only apparent during the third trimester, while had limited significance during the early-pregnancy. H1 accounts for the highest proportion (81.6%) with no significant growth in changes during different pregnancy periods, but there was a gradient difference when compared with H2 and H3. It was also worth noting that 1.8% of pregnant women(H4) had low levels of HOMA-IR in the early and middle stages of pregnancy, similar to H1, but significantly increased in the late stages of pregnancy even than H2 and H3.

For QUICKI, a long-term declining trend was observed over time, representing the gradually incremental insulin resistance levels. The trajectory 2(Q2) of QUICKI, with the maximum proportion, showed a relatively stable pattern of index changes from mid to late pregnancy. Conversely, trajectory 4 (Q4) of QUICKI displayed a reverse upward trend, suggesting a modest increase in insulin sensitivity within a narrow range of amplitudes.

### 3.3 Association between gestational trajectories and perinatal complications

As shown in **Table 2**, after adjusting for potential confounding factors, the incidence of perinatal complications exhibited an upward trend with the increasing severity of IR across different trajectory subgroups of FINS. Specifically, the incidence of adverse pregnancy outcomes was significantly higher in F3 compared with F1 (aRR, 1.35 [95% CI, 1.10–1.65]; P = 0.004). A similar trend was observed in H2, which demonstrated a marginally elevated incidence relative to H1 (aRR, 1.27 [95% CI, 1.08–1.50]; P = 0.004). In contrast, the incidence of perinatal complications was notably lower in the Q2, Q3, and Q4 subgroups of the QUICKI trajectories than in Q1 (Q2: aRR, 0.79 [95% CI, 0.69–0.92], P<0.002; Q3: aRR, 0.68 [95% CI, 0.46–0.99], P = 0.049; Q4: aRR, 0.68 [95% CI, 0.52–0.90], P = 0.007).

**Table 2:** Associations of insulin resistance trajectories with perinatal complications.

Insulin resistance trajectories		Model I	P-value	Model II	P-value	Model III	P-value
Adjusted relative risk (95% CI)							
FINS	Trajectory 1: F1	*1 (Reference)*		*1 (Reference)*		*1 (Reference)*	
	Trajectory 2: F2	1.26 (1.09,1.45)	0.04	1.15 (0.99,1.35)	0.07	1.15 (0.99,1.35)	0.07
	Trajectory 3: F3	1.39 (1.14,1.68)	<0.001	1.34 (1.10,1. 64)	0.004	1.35 (1.10,1. 65)	0.004*
	Trajectory 4: F4	1.43 (0.96,2.13)	0.07	1.42 (0.95,2.12)	0.09	1.42 (0.96,2.08)	0.08
HOMA-IR	Trajectory 1: H1	*1 (Reference)*		*1 (Reference)*		*1 (Reference)*	
	Trajectory 2: H2	1.31 (1.13,1.54)	0.001	1.30 (1.09,1.50)	0.003	1.27 (1.08,1.50)	0.004*
	Trajectory 3: H3	1.12 (0.72,1.74)	0.61	1.10 (0.72,1.70)	0.66	1.10 (0.71,1.71)	0.67
	Trajectory 4: H4	1.20 (0.78,1.85)	0.39	1.20 (0.78,1.85)	0.40	1.21 (0.80,1.83)	0.38
QUICKI	Trajectory 1: Q1	*1 (Reference)*		*1 (Reference)*		*1 (Reference)*	
	Trajectory 2: Q2	0.78 (0.68,0.89)	<0.001	0.80 (0.69,0.92)	0.002	0.79 (0.69,0.92)	0.002*
	Trajectory 3: Q3	0.67 (0.46,0.97)	0.03	0.69 (0.48,0.99)	0.049	0.68 (0.46,0.99)	0.049
	Trajectory 4: Q4	0.67 (0.51,0.89)	0.005	0.69 (0.52,0.91)	0.008	0.68 (0.52,0.90)	0.007*

FINS, fasting Insulin; HOMA-IR, homeostasis model assessment of insulin resistance; QUICKI, quantitative insulin sensitivity check index.

Perinatal complications including gestational diabetes mellitus, hypertensive disorders of pregnancy, preterm birth, dystocia, postpartum hemorrhage, neonatal admission to neonatal intensive care, small-for-gestational age, large-for-gestational age and macrosomia.

Data are presented as adjusted relative risk (95% confidence interval) calculated using Poisson regression models with robust standard errors.

P-value was calculated using the Poisson trend test. *P<0.05, adjusted for multiple testing using the Benjamini-Hochberg (BH) false discovery rate (FDR) correction in model 3.

Model 1 was unadjusted.

Model 2 adjusted for age at delivery, nulliparity, pre-pregnancy BMI, assisted reproduction.

Model 3 adjusted for factors in Model 2 in addition to fasting glucose levels in early-pregnancy, GDM history, and family history of diabetes.

In the analysis of trajectory modeling and preliminary correlation,the classification of FINS was found to possess considerable clinical significance and exhibit statistically significant variations. Therefore, we proceeded to conduct a comprehensive analysis and exploration of the four trajectories of FINS (**Table 3**). Since F1 showed the lowest insulin level to meet the physiological requirements, it generally indicated the highest insulin sensitivity and the healthiest physiological state, thus we adopted it as the reference trajectory. In model 3, the gradient of associations across FINS trajectories (from F1 to F3) with GDM and HDP were observed. Specifically, the risk of GDM increased across F1 to F3, with F2 showing a moderately elevated association (aRR, 1.73 [95% CI, 1.21-2.47]; P for trend<0.01) and F3 showing a more pronounced association (aRR, 2.40 [95% CI, 1.58-3.65]; P for trend< 0.001). Similarly, the risk of HDP exhibited an increasing trend from F1 to F4, with F3 showing a moderate association (HDP: aRR, 5.86 [95% CI, 2.41–14.25]; P for trend< 0.001) and F4 showing a stronger association (HDP: aRR, 6.45 [95% CI, 1.78–23.33]; P for trend< 0.001).

**Table 3:** Association of gestational serum insulin trajectories with perinatal complications using the low insulin level trajectory as reference group.

Perinatal complications	F1	F2	F3	F4	*P*-value
	Adjusted relative risk (95% CI)				
GDM					
Model I	*1 (Reference)*	1.83 (1.28,2.61)^a^	2.75 (1.84,4.12)^a^	1.71 (0.65,4.47)	<0.001
Model II	*1 (Reference)*	1.78 (1.25,2.54)^a^	2.54 (1.68,3.86)^a^	1.70 (0.65,4.45)	<0.001
Model III	*1 (Reference)*	1.73 (1.21,2.47)^b^	2.40 (1.58,3.65)^a^	1.54 (0.58,4.09)	<0.001
HDP					
Model I	*1 (Reference)*	2.17 (0.96,4.92)	6.87 (3.01,15.70)^a^	6.40 (1.75,23.46)^b^	<0.001
Model II	*1 (Reference)*	2.12 (0.94,4.77)	5.79 (2.46,13.63)^a^	6.06 (1.64,22.36)^b^	<0.001
Model III	*1 (Reference)*	2.11 (0.94,4.77)	5.86 (2.41,14.25)^a^	6.45 (1.78,23.33)^a^	<0.001
PPH					
Model I	*1 (Reference)*	1.30 (0.86,1.97)	0.90 (0.48,1.69)	2.06 (0.78,5.46)	0.255
Model II	*1 (Reference)*	1.31 (0.87,1.97)	0.84 (0.46,1.52)	1.97 (0.73,5.31)	0.183
Model III	*1 (Reference)*	1.37 (0.91,2.08)	0.93 (0.50,1.72)	2.01 (0.74,5.47)	0.199
LGA& Macrosomia					
Model I	*1 (Reference)*	0.97 (0.57,1.65)	1.80 (0.98,3.34)	0.75 (0.10,5.37)	0.127
Model II	*1 (Reference)*	0.96 (0.57,1.64)	1.69 (0.89,3.21)	0.73 (0.11,5.10)	0.270
Model III	*1 (Reference)*	0.99 (0.59,1.66)	1.79 (0.94,3.41)	0.73 (0.10,5.12)	0.227
SGA					
Model I	*1 (Reference)*	1.08 (0.63,1.84)	0.53 (0.20,1.39)	0.79 (0.11,5.67)	0.499
Model II	*1 (Reference)*	1.08 (0.63,1.84)	0.58 (0.21,1.60)	0.82 (0.11,5.92)	0.627
Model III	*1 (Reference)*	1.14 (0.67,1.96)	0.67 (0.24,1.91)	0.95 (0.13,6.74)	0.734
NICU					
Model I	*1 (Reference)*	1.19 (0.75,1.90)	0.93 (0.46,1.89)	1.46 (0.37,5.71)	0.780
Model II	*1 (Reference)*	1.17 (0.73,1.89)	0.88 (0.40,1.95)	1.44 (0.37,5.70)	0.772
Model III	*1 (Reference)*	1.17 (0.73,1.89)	0.86 (0.39,1.93)	1.43 (0.37,5.51)	0.756

GDM, gestational diabetes mellitus; HDP, hypertensive disorders of pregnancy; PPH, postpartum hemorrhage; AGA, appropriate-for-gestational age; LGA, large-for-gestational age; SGA, small for gestational age; NICU, neonatal intensive care unit; T1–T4, trajectories 1–4 of serum insulin level.

Data are presented as adjusted relative risk (95% confidence interval) calculated using Poisson regression models with robust standard errors.

P-for-trend was calculated using the Poisson trend test.

Model 1 was unadjusted.

Model 2 adjusted for age at delivery, pregnancy, nulliparity, pre-pregnancy BMI, assisted reproduction.

Model 3 adjusted for factors in Model 2 in addition to higher early-pregnancy glucose levels, GDM history, and family history of diabetes.

^a^P<0.001; ^b^P<0.01;^c^P<0.05.

^d^LGA, SGA: Birth weight categories derived using gestational age-specific percentiles calculated using a 2019 Chinses reference population.

In the HOMA-IR trajectories (**Supplementary Table S3**), H1 exhibited stable low levels of insulin resistance across the entire gestational period, with a maximum prevalence of 81.6%. Based on this, we selected it as the control subgroup. After sufficient adjustment for relevant confounding factors, there were still significant differences in the risk of GDM, HDP, and LGA among subgroups. For GDM, a moderately increased association was observed in H2 (aRR, 1.61 [95% CI, 1.19–2.61]; P<0.01), while H3 demonstrated a higher relative risk in the unadjusted model (aRR, 2.00 [95% CI, 1.13–3.97]; P<0.05), which did not reach statistical significance after adjusted. Additionally, H2 was associated with an elevated risk of HDP (aRR, 2.70 [95% CI, 1.43–5.10]; P<0.01). H4 showed a marked increase in late pregnancy, and was associated with the highest risk of HDP (aRR, 4.31 [95% CI, 1.36–13.70]; P<0.01). Notably, H3 did not exhibit a statistically significant association with HDP, but was linked to an increased risk of LGA and macrosomia (aRR, 3.18 [95% CI, 1.25–8.05]; P<0.01).

In a similar vein, although QUICKI differed from the aforementioned two indices due to its negative correlation, it remained worthy of exploration and analysis. Among the QUICKI trajectories, Q1 was identified as a subgroup with significant IR throughout early, middle, and late pregnancy, which was distinct from other subgroups characterized by stable or moderate insulin resistance. Considering the negative correlation characteristics of the index itself, we listed it as a control. After adjusted, we observed significant statistical differences in the risk of GDM and HDP.

As presented in **Supplementary Table S4**, Q2 was associated with a reduced incidence of GDM (aRR, 0.61 [95% CI, 0.47-0.80]; P<0.001), whileQ4 exhibited a more pronounced reduction in GDM risk (aRR, 0.42 [95% CI, 0.23-0.78]; P<0.01). At the same time, a gradient decrease in the association with HDP risk was observed from Q1 to Q4, with Q2 showing a moderate reduction in risk (aRR, 0.34 [95% CI, 0.19-0.62]; P<0.001), Q3 continuing to decline based on this (aRR, 0.30 [95% CI, 0.14-0.58]; P<0.01), and Q4 exhibiting a lower incidence rate (aRR, 0.22 [95% CI, 0.05-0.87]; P<0.05).

To verify the robustness of our findings, sensitivity analyses were conducted. 1) We incorporated the posterior probabilities of HOMA-IR trajectory membership derived from the group-based trajectory model into the Poisson regression model instead of using a hard classification of participants into discrete trajectory groups (**Supplementary Table S5** in the **Supplementary Materials**). The posterior probabilities were treated as continuous predictors to minimize potential bias from classification error. The results of sensitivity analyses were generally consistent with those of the main analysis. 2) We use logistic regression model with the summary IR metrics (including cumulative measurement, time-weighted Average IR, etc). In **Supplementary Table S6**, the results showed that most of these summary IR metrics were significantly associated with GDM and HDP risks, and the direction of the associations was consistent with our primary analysis. Only a few metrics (e.g., Last measurement in association with GDM, some standard deviation) showed no significant associations, which did not affect the robustness of our main conclusions. 3)As FINS is the most concerned indicator in this article, we conducted the further sensitivity analysis. We use linear mixed model to estimate each subject’s intercept and slope of IR trajectories, then incorporate these parameters into logistic regression for outcome risk prediction. The results confirm that a faster increase in IR over time is a consistent risk factor for GDM (OR = 1.007, 95% CI: 1.005-1.009, P< 0.001) and HDP (OR = 1.011, 95% CI: 1.007-1.015, P< 0.001), while baseline IR level plays a less prominent or different role depending on the outcome.

### 3.4 Association between individual-level multidomain factors and serum insulin trajectories

Based on the IR index FINS, which was directly tested by pregnant women’s serum, we conducted Poisson regression analysis on the correlation between individual level multiple factors and IR subtypes. As shown in **Supplementary Table S5**, in the adjusted covariate model, the proportion of elderly, overweight and obese pregnant women appearing in the F4 trajectory was higher. At the same time, the risk of overweight or obese pregnant women in the F3 subgroup was also significant, while the probability of lean pregnant women appearing in the F1 subgroup was significantly higher than that in F3 or F4 (P<0.05). Compared with women in the F1 subgroup, there was a higher risk of developing F2 or F3 trajectories with higher fasting blood glucose levels (5.1-6.9 mmol/L) during the initial prenatal examination. However, women with diabetes history of family first-degree relatives were more likely to have the trend of F3 or F4 track changes during pregnancy (P<0.05).

### 3.5 The risk stratification ability of IR indices in early pregnancy

**Figure 2:**
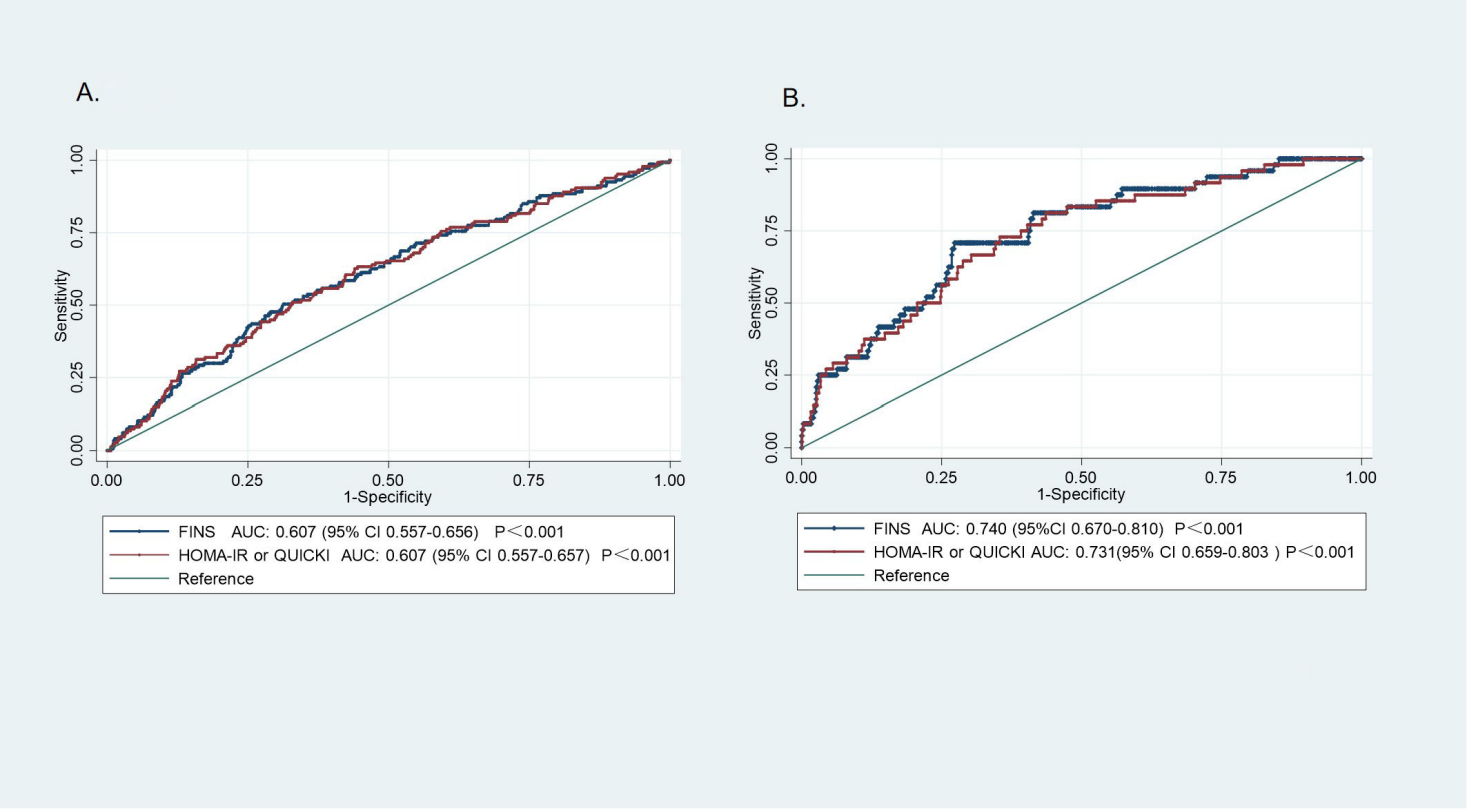
Receiver operating characteristic (ROC) curve for risk stratification of perinatal complications using early-pregnancy IR indices. **(A)** GDM, **(B)** HDP. GDM, gestational diabetes mellitus; HDP, hypertensive disorders of pregnancy; AUC, Area Under Curve.

We assessed the risk stratification capacity of IR indices in early pregnancy by examining their association with adverse outcomes diagnosed in later gestation (**Figure 2**). Their discriminatory performance is presented in **Table 4**. For GDM diagnosed by OGTT in mid-pregnancy, IR indices measured in early pregnancy demonstrated a limited capacity to stratify women at risk. FINS achieved an AUC of 0.607 (P<0.001). Due to their mathematical interdependence, HOMA-IR and QUICKI produced identical discriminatory results, with completely overlapping ROC curves (AUC 0.607, P<0.001). However, these indices exhibited a more robust capacity for stratifying the potential risk of HDP diagnosed in the second and third trimesters. FINS showed the highest discriminatory ability, with an AUC of 0.740 (P<0.001). At an optimal cutoff of 7.68, it identified at-risk individuals with a sensitivity of 70.83% and specificity of 72.71%. HOMA-IR and QUICKI demonstrated slightly lower discriminatory performance (AUC 0.731), with cutoff values of 1.20 and 0.37, respectively, yielding a sensitivity of 81.25% and specificity of 56.41%.

**Table 4:** The predictive efficacy of IR indicators in early pregnancy.

Outcome	Index	AUC	95%CI	Z-value	SE	P	Cut-off	Sensitivity	Specificity
GDM	FINS	0.607	0.577-0.636	4.198	0.025	<0.001	7.28	50.34	68.66
	HOMA-IR	0.607	0.577-0.636	4.201	0.025	<0.001	1.17	63.27	55.54
	QUICKI						0.374		
HDP	FINS	0.740	0.713-0.766	6.720	0.036	<0.001	7.68	70.83	72.71
	HOMA-IR	0.731	0.703-0.757	6.300	0.037	<0.001	1.20	81.25	56.41
	QUICKI						0.372		

GDM, gestational diabetes mellitus; HDP, hypertensive disorders of pregnancy; AUC, Area Under Curve; SE, standard error.

## 4 Discussion

In this study involving 1504 pregnant women, we identify the trends and potential subtypes of IR changes throughout early, middle, and late pregnancy. As the gestational age progresses, the degree of IR during pregnancy gradually increases, but it is worth noting that IR gradient differences may occur in early pregnancy and persist until final delivery.

The application of latent class model in trajectory analysis is a creative semiparametric statistical approach for identifying distinct trends over time in longitudinal data (20).The four-trajectory classification was able to distinguish between mild, moderate, severe, and special IR changes across pregnancy, which is more applicable for clinical risk stratification compared with fewer or more trajectory models. Prior research on FINS has identified diagnostic criteria for hyperinsulinemia and IR classification in non-pregnant adults (21), but these criteria are currently absent in pregnant individuals. In this regard, our findings contribute to the literature by demonstrating the direct correlations between the risk of perinatal complications and gestational IR trajectories.

Previous studies have shown that accurately assessing maternal pancreatic beta cell function, insulin sensitivity, or IR is of great significance for the health of pregnant women. In the study, we mainly select FINS, HOMA-IR, and QUICKI for IR assessment. Our study shows that the FINS levels of most pregnant women in the FINS fitted trajectory gradually increase slowly with the increase of gestational age, exhibiting good gradient differences from the beginning of early pregnancy. 1.9% of pregnant women show a special trajectory, with baseline levels and growth rates in early to mid pregnancy being close to the median level (54.9%), which accounts for the majority of the population, then shows a significant increase during mid to late pregnancy and reached the highest insulin resistance level among pregnant women in late pregnancy. Some studies have shown that the gradually enhanced insulin secretory response in early pregnancy occurs prior to changes in insulin resistance during gestation and persists alongside the progression of insulin resistance (2, 22). This suggests that when β-cell compensation initially becomes ineffective, insulin secretion is continuously promoted, which is consistent with the observed pattern in our trajectory plots. Therefore, FINS in early pregnancy may be a more sensitive indicator than other assessment indices. It is worth noting that F4 indicates the presence of a small proportion of pregnant women with special trajectories that only significantly increases in the late pregnancy. This may be related to the population with stable pregnancy but sudden onset of severe complications (such as severe gestational hypertension) in late pregnancy. The reason for the phenomenon may be related to changes in placental function in the late stage that induces different IR factors or changed maternal susceptibility to these factors.

After adjustment, pregnant women with higher levels of FINS in early and middle pregnancy were associated with a 2.4-fold higher risk of GDM compared with those with lower FINS. Our findings indicate that insulin levels in early and middle pregnancy are positively associated with GDM risk, which is consistent with previous reports by Mittendorfer, Grewal, and others (1, 23). In addition, in analyses of HDP, a gradient increase in risk was observed across F1 to F4, suggesting that elevated insulin levels were also independently associated with HDP. Compared with the risk of GDM, the risk of HDP in F4 was three times higher than that in F2, suggesting that the association of IR during pregnancy with HDP was present across the entire gestational period. Therefore, during pregnancy, attention to and management of IR should not be limited to a specific time point or period, but should be maintained throughout early, middle, and late pregnancy. Previous studies on the relationship between serum insulin and HDP have been limited. Future studies based on FINS trajectories may help to further clarify the potential benefits of insulin management in reducing the risk of adverse pregnancy complications.

It is worth noting that in HOMA-IR trajectories, 1.8% of the population has trajectories that almost overlaps with the low subgroup with the largest sample proportion in early and middle pregnancy, but ultimately grows to the highest trajectory point of the study population. It means that this population is monitored by both classification methods, which reduces the possibility of method errors. Special trajectories only shows significant differences from low level subgroups in late pregnancy, but whether there are differences in the characteristics and pregnancy outcomes of the two subgroups is worth further exploration. The poor discriminability of HOMA-IR trajectories in early and middle pregnancy may be related to the smaller data values obtained by HOMA-IR calculation methods, as well as the potising predictive performance of HOMA-IR in early pregnancy. The non-significant changes in the early and middle pregnancy period also appeares in the study of Skajaa et al., and their statistical results showed that the insulin demand of pregnant women with diabetes before 22 weeks of pregnancy only increased by 9%, while it could increase by more than 70% in the near term pregnancy (24).

The risk of GDM was found to increase by more than 60% across HOMA-IR trajectories above the population mean, with a gradient increase observed from H1 to H3. For subgroups with a significant increase only in late pregnancy, the association with GDM risk did not reach statistical significance, which further highlights the importance of insulin resistance changes during early and middle pregnancy. In addition, H4 was associated with the highest risk of HDP among the four subgroups, suggesting an association between IR in late pregnancy and HDP occurrence. Meanwhile, the risk of LGA or macrosomia showed a gradual increase in H2 and H3, but not in H4, indicating that the association between IR and fetal growth and development may already be present during early and middle pregnancy. These findings are consistent with previous research results (25–27). It is worth noting that this trend is only found in HOMA-IR, we consider that FINS reflects more insulin secretion response, while HOMA-IR includes both fasting insulin and fasting blood glucose parameters, based on the interaction between blood glucose and insulin in different tissues and organs (including pancreas, liver, and surrounding tissues) (28).Previous studies have suggested that higher HOMA-IR was significantly related to the increase of LGA incidence rate, and the risk of HOMA-IR increased by 1.5 times every additional unit (29). Similarly, in Dinh et al.’s study (30), there was a significant correlation between HOMA-IR and fetal ultrasound weight estimation during mid pregnancy, but not in QUICKI. It may suggest that HOMA-IR have an advantage in predicting fetal growth, development, and adverse outcomes.

Although QUICKI and HOMA-IR include the same calculation variables, comparing that HOMA-IR is a non-normal distribution parameter, QUICKI undergo logarithmic transformation during the improvement process, and ultimately shows a negative correlation with insulin resistance. In early pregnancy, maternal blood glucose undergoes a physiological decline (to meet the early developmental needs of the fetus) (31). At this stage, the magnitude of change in fasting glucose is relatively small, making it difficult to reflect subtle abnormalities in early insulin resistance. But HOMA-IR and QUICKI are calculated from fasting glucose and fasting insulin. When glucose is at a physiologically low level and exhibits minimal fluctuation, changes in these indices are diluted by glucose, thereby failing to accurately reflect abnormalities at the insulin level.

In QUICKI, there is a small proportion of the population (9.6%) whose degree of IR during pregnancy tends to be stable with little change, which may be closely related to weight gain, dietary control, physical activity, and good maternal beta cell function (31–33). The population characteristics and final pregnancy outcomes of this group of people are worthy of further tracking and exploration.

In addition, when the trajectory with the highest degree of IR is used as a control, the incidence of GDM and HDP in the sensitive subgroup significantly decreases. However, similar to H3 and H1 in HOMA-IR, including the comparison between Q3 and Q1, the association difference of GDM between the two still exists after the adjustment of pre-pregnancy BMI, age and other factors. However, when further hybrid adjustment is made for high fasting blood glucose in early pregnancy, GDM history, and diabetes in the family’s first degree relatives, the differential risk of GDM disappears, which may indicate that when QUICKI and HOMA-IR both include fasting blood glucose as a calculation parameter, adjusting the risk of gestational glucose metabolism disease in the population with high fasting blood glucose in early pregnancy will have an impact on the steady-state model, corresponding to the proposed limitations of HOMA-IR in the population with significantly damaged islet beta cells or abnormal glucose (34). For HDP, a gradually decreasing gradient risk is observed in Q1-Q4, but Q4 remains stable throughout pregnancy in the special trajectory, and shows the lowest HDP risk, only 22% of the high IR subgroup population. Further population factor analysis of Q4 population characteristics may have certain clinical value for long-term IR management and prevention.

In this study, pregnant women who received pharmacological treatment (metformin or insulin) for hyperglycemia were excluded. Consequently, individuals with more severe glucose dysregulation were underrepresented in the analytical sample, which may have attenuated the observed associations with GDM. Therefore, the late-pregnancy IR trajectories (e.g., F4, H4) observed in our study may underestimate the actual severity of IR in GDM patients without intervention. Considering that the diagnosis of GDM in our hospital is concentrated between 24–28 weeks of pregnancy, and once GDM is diagnosed in clinical, a multidisciplinary team will immediately develop personalized treatment plans for patients, including dietary control, appropriate regular exercise, and insulin intervention. Therefore, changes in IR during early and mid pregnancy have significance for the risk of GDM, while changes during late pregnancy provide more clues to the risk of complications and prognosis. In fact, previous studies had found that even if the recommended diagnosis and treatment plan was followed well, some GDM patients still cannot improve GDM complications, which can be attributed to the IR subtype (5, 35). The heterogeneity of IR in GDM pregnant women to some extent led to different pregnancy outcome events (36, 37). Therefore, depth exploration of the relationship between changes in IR trajectory during pregnancy and pregnant complications may help identify women at high risk of adverse outcomes, which can be managed in early pregnancy or even before pregnancy, and supplement insulin sensitization therapy if necessary to improve prognosis.

Women in high insulin resistance trajectories exhibited a significantly increased risk of HDP, with magnitudes varying by indicator: those with high FINS levels faced a nearly six-fold higher risk, while women with high HOMA-IR or low QUICKI levels had approximately three-fold higher risks. These findings suggest that the IR patterns emerging in early pregnancy may help identify women at elevated risk for HDP in later pregnancy, a finding consistent with previous studies (5). It is worth noting that in our model, only special trajectory subtypes that shows significant increases in late pregnancy, such as F4 classified by FINS or H4 classified by HOM-IR, both shows higher risk values for HDP than those with normal high resistance levels. This may indicate that IR in late pregnancy is a related influencing factor and participates in the pathological and physiological processes of HDP occurrence and development. Although cardiovascular diseases such as hyperinsulinemia, IR, and primary hypertension are popular research areas in non-pregnant populations (38–40), studies in pregnant populations mostly focus on group observation and control at a single stage. This study is the first to propose the correlation between different pregnancy stages, IR dynamic changes, and HDP. The mechanism that hyperinsulinemia and IR may induce pregnancy hypertension is still unclear, although there are some hypothetical inferences: for example, IR affects angiogenesis pathway and mediates vascular endothelial damage (41), hyperinsulinemia inhibits sodium retention caused by sodium transport in proximal renal tubules (42), hyperinsulinemia and increased muscle sympathetic nerve activity caused by plasma norepinephrine (43), etc.

Previous studies have shown that multiple factors at the individual level, such as age and weight, are closely related to IR levels (44, 45). In the analysis of multiple factors and IR trajectories at the individual level, compared with F1 individuals, we found that women who are overweight, obese, and have higher fasting blood glucose levels in early pregnancy are more likely to appear on trajectories with higher IR levels. However, women with a history of diabetes in their immediate family and elder age groups may also have IR in early and middle pregnancy, which is close to that of ordinary people, and only increases significantly in the late pregnancy. The results of this study provide a better understanding of IR subgroups that follow different IR change trajectories and their individual level characteristics. This may allow for risk stratification of high-risk pregnancies (46) and provide information for future multi-component interventions to reduce the risk of perinatal complications. Specifically, our research results can support efforts to address modifiable risk factors, such as overweight and obesity management in the reproductive age population, as well as high-frequency outpatient or telephone follow-up and education for high-risk pregnant women.

This study demonstrates that FINS in early pregnancy exhibits a moderate discriminatory ability for HDP with an AUC of 0.740, and the optimal cutoff value was identified as 7.68. Following sensitivity analyses adjusting for confounders such as BMI and age, this threshold remained applicable in pregnant women with normal BMI or those of non-advanced maternal age. Notably, previous studies have lacked established cutoff values for FINS in relation to HDP, with research primarily focusing on its utility for GDM. A cohort study involving 298 pregnant women in India reported that an early pregnancy FINS cutoff of >7.45 μU/mL yielded 80% sensitivity and 57.4% specificity for GDM (23), which aligns closely with the optimal GDM cutoff of 7.28 identified in our study. Additionally, Bitó et al., in a study of 75 European pregnant women with multiple GDM risk factors, found that FINS measured before 16 weeks of gestation achieved 96.4% specificity for GDM risk stratification (47). However, other studies have reported that FINS did not demonstrate superior discriminatory performance compared to other IR indicators (48).

At the same time, HOMA-IR and QUICKI also demonstrate moderate capacity for risk stratification in early pregnancy, slightly inferior to the former with an AUC of 0.731 and cutoff values of 1.20 and 0.37, respectively. A multicenter cohort study based on 700 pregnant women in China set the 75th percentile of the population at 1.76 as the cutoff value (49), and another prospective cohort of 1343 pregnant women in Beijing proposed that the optimal cutoff value for risk stratification for GDM was 1.52 (AUC value 0.733, sensitivity 63%, specificity 71%) (46). Meanwhile, a prospective study in India (23) showed that the risk of GDM significantly increased when the HOMA-IR value was ≥ 1.17 (sensitivity 73.3%, specificity 61.6%), or the QUICKI value was ≤ 0.35 (sensitivity 62.3%, specificity 68.7%), which is similar to the cutoff value of our study. Considering that various factors such as race and living environment have an impact on HOMA-IR (50), the diagnostic threshold values proposed based on research on European women are mostly greater than 2 (44, 51–54), showing a trend higher than Asian women. Therefore, we need prospective, multicenter large sample studies to further fully demonstrate this. In addition, although most studies on QUICKI have been conducted simultaneously with HOMA-IR, some scholars believe that it can exhibit stronger specificity in risk stratification for GDM.

### 4.1 Strengths and limitations

The strengths of this study include a longitudinal design that distinctly shows IR profiles throughout pregnancy. Additionally, the study employs GBTM to analyze the underlying patterns within the longitudinal data. To the best of our knowledge, this study represents the first Chinese population-based investigation that aims to characterize the patterns of IR during different stages of pregnancy and their association with the likelihood of experiencing adverse pregnancy outcomes. Nevertheless, it is important to acknowledge the limitations of this study. Firstly, this is a retrospective observational study, and all identified relationships between IR trajectories and adverse pregnancy outcomes are associative rather than causal. Further prospective interventional studies are needed to verify potential causal links. Second, despite our efforts to control for various covariates, some confounding factors may still exist, such as individuals’ gestational weight gain, dietary pattern, and maternal exercise during pregnancy, which were previously reported to have an impact on glycolipid metabolism and perinatal outcomes. Moreover, individuals with prediabetes and hyperinsulinemia are not excluded, as prediabetes with earlier glucose control may serve as precursors to gestational dysglycemia, as per the guidelines set by the American Diabetes Association (ADA) (55). Future research endeavors should focus on addressing these areas in need of enhancement.

## 5 Conclusions

There are various subtypes of IR trajectories in pregnant women, which are significantly correlated with adverse pregnancy outcomes. The risk of GDM and HDP increases gradually from low level trajectories to high trajectories. The IR levels during early and middle pregnancy are closely related to the occurrence of GDM, while the risk of HDP is influenced by IR levels throughout the entire pregnancy period. The FINS values during early pregnancy, along with the HOMA-IR and QUICKI evaluation index, can be utilized to predict the risk of GDM and HDP. Further research endeavors focusing on elucidating the underlying mechanism of hyperinsulinemia and insulin resistance will provide valuable insights for early clinical interventions.

## Data Availability

The raw data supporting the conclusions of this article will be made available by the authors, without undue reservation.
