# Supplementary material for: Association between insulin resistance trajectories and perinatal complications: a retrospective cohort study
Source: Front Endocrinol (Lausanne). 2026 Mar 30;17:1746255. doi: 10.3389/fendo.2026.1746255 (PMC13070787; doi:10.3389/fendo.2026.1746255)
Supplement: Supplementary file 1 [file DataSheet1.docx]

Supplementary Material

# Supplementary Figures and Tables

For more information on Supplementary Material and for details on the different file types accepted, please see [here](https://www.frontiersin.org/guidelines/author-guidelines#supplementary-material).

## Supplementary Figures


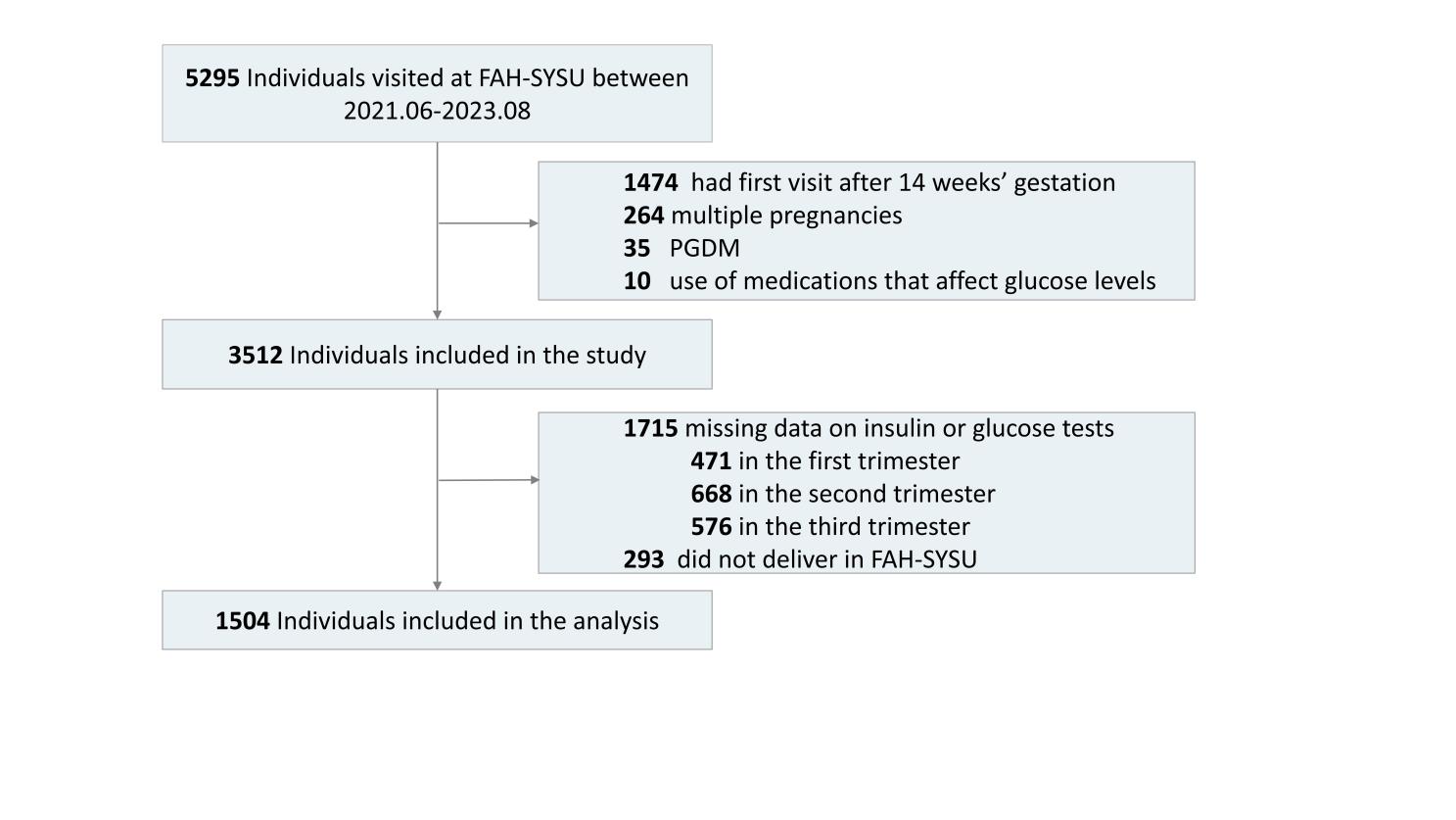


**Supplementary eFigure 1.**The study cohort selected by the inclusion and exclusion criteria. FAH- SYSU, the First Affiliated Hospital of Sun Yat-sen University; PGDM, pregestational Diabetes Mellitus

- 1. **Supplementary Tables**

| **eTable 1. Trajectory Modeling Fit Criteria** | | | | | | | |
| --- | --- | --- | --- | --- | --- | --- | --- |
|  |  |  |  | **Posterior predictive probability** | | | |
|  | **Groups** | **BIC** | **AIC** | **Mean** | **SD** | **Minimum** | **Maximum** |
| **Serum insulin trajectories** | 4 | 980.95 | 1029.06 | 0.88 | 0.05 | 0.85 | 0.95 |
| **HOMA-IR trajectories** | 4 | 5151.04 | 5102.93 | 0.96 | 0.05 | 0.89 | 1.00 |
| **QUICKI trajectories** | 4 | 9375.37 | 9420.27 | 0.82 | 0.03 | 0.79 | 0.85 |
| Abbreviations: HOMA-IR, homeostasis model assessment of insulin resistance; QUICKI, quantitative insulin sensitivity check index; BIC, Bayesian information criterion; AIC, Akaike information criterion. | | | | | | | |

**eTable 2. Selection of Group Number and Subgroup Order for Trajectory Modeling**

| **Trajectory** | **Classification** | **Group Order** | **Subgroup** | | | | | **BIC**  **N=1504** |
| --- | --- | --- | --- | --- | --- | --- | --- | --- |
|  |  |  | **1** | **2** | **3** | **4** | **5** |  |
| **FINS** | 2 | 2,1 | 0.92 | 0.87 |  |  |  | 556.90 |
|  | 3 | 2,1,2 | 0.92 | 0.88 | 0.96 |  |  | 662.54 |
|  | 4 | 2,2,1,2 | 0.86 | 0.85 | 0.83 | 0.97 |  | 748.38 |
|  | 5 | 2,2,1,1,2 | 0.82 | 0.81 | 0.83 | 0.86 | 0.97 | 766.74 |
| **HOMA-**  **IR** | 2 | 1,2 | 0.99 | 0.99 |  |  |  | 5939.52 |
|  | 3 | 2,1,1 | 0.99 | 0.99 | 0.93 |  |  | 5358.18 |
|  | 4 | 1,2,2,2 | 0.89 | 0.98 | 0.97 | 0.99 |  | 5142.80 |
|  | 5 | 1,2,2,2,2 | 0.87 | 0.94 | 0.96 | 0.98 | 0.99 | 5027.33 |
| **QUICKI** | 2 | 1,2 | 0.89 | 0.86 |  |  |  | 9276.59 |
|  | 3 | 1,2,2 | 0.84 | 0.83 | 0.82 |  |  | 9349.86 |
|  | 4 | 1,2,2,1 | 0.85 | 0.79 | 0.84 | 0.79 |  | 9383.06 |
|  | 5 | 1,1,1,1,2 | 0.83 | 0.79 | 0.84 | 0.79 | 0.99 | 9416.83 |

Abbreviations: FINS,fasting serum insulin; HOMA-IR, homeostasis model assessment of insulin resistance; QUICKI, quantitative insulin sensitivity check index; BIC, Bayesian information criterion

**eTable 3 Association of Gestational HOMA-IR Trajectories With Perinatal Complications Using the Low HOMA-IR Level Trajectory as Reference Group**

| **Perinatal complications** | **H1** | **H2** | **H3** | **H4** | ***P*-value** |
| --- | --- | --- | --- | --- | --- |
|  | Adjusted relative risk (95% CI) | | | |  |
| **GDM** |  |  |  |  |  |
| Model Ⅰ | *1（Reference）* | 1.95(1.47,2.59)^a^ | 2.00(1.13,3.97)^c^ | 0.61(0.16,2.34) | ＜0.001 |
| Model Ⅱ | *1（Reference）* | 1.69(1.25,2.27)^a^ | 1.91(1.12,3.78)^c^ | 0.61(0.16,2.36) | 0.002 |
| Model Ⅲ | *1（Reference）* | 1.61(1.19,2.16)^b^ | 1.85(0.93,3.67) | 0.58(0.16,2.18) | 0.006 |
| **HDP** |  |  |  |  |  |
| Model Ⅰ | *1（Reference）* | 3.75(2.26,6.22)^a^ | 2.43(0.61,9.62) | 3.91(1.38,11.94)^b^ | ＜0.001 |
| Model Ⅱ | *1（Reference）* | 2.72(1.48,5.00)^a^ | 2.13(0.60,7.64) | 3.97(1.25,12.58)^b^ | 0.004 |
| Model Ⅲ | *1（Reference）* | 2.70(1.43,5.10)^b^ | 2.04(0.58,7.21) | 4.31(1.36,13.70)^b^ | 0.006 |
| **PPH** |  |  |  |  |  |
| Model Ⅰ | *1（Reference）* | 0.67(0.38,1.21) | 0.42(0.06,2.91) | 1.80(0.72,4.55) | 0.234 |
| Model Ⅱ | *1（Reference）* | 0.59(0.33,1.04) | 0.38(0.06,2.49) | 1.67(0.65,4.28) | 0.106 |
| Model Ⅲ | *1（Reference）* | 0.62(0.35,1.10) | 0.41(0.06,2.67) | 1.67(0.64,4.33) | 0.158 |
| **LGA&**  **Macrosomia** |  |  |  |  |  |
| Model Ⅰ | *1（Reference）* | 1.94(1.16,4.24)^c^ | 3.21(1.24,8.27)^c^ | 0.86(0.12,5.99) | 0.039 |
| Model Ⅱ | *1（Reference）* | 1.58(0.89,2.80) | 2.95(1.17,7.43)^c^ | 0.82(0.12,5.34) | 0.074 |
| Model Ⅲ | *1（Reference）* | 1.69(0.94,3.00) | 3.18(1.25,8.05)^c^ | 0.81(0.12,5.38) | 0.049 |
| **SGA** |  |  |  |  |  |
| Model Ⅰ | *1（Reference）* | 0.49(0.20,1.21) | 0.53(0.20,1.39) | 0.78(0.11,5.47) | 0.808 |
| Model Ⅱ | *1（Reference）* | 0.64(0.25,1.64) | 0.53(0.19,0.40) | 0.83(0.12,5.83) | 0.636 |
| Model Ⅲ | *1（Reference）* | 0.70(0.27,1.83) | 0.53(0.20,1.39) | 0.87(0.12,6.13) | 0.763 |
| **NICU** |  |  |  |  |  |
| Model Ⅰ | *1（Reference）* | 0.95(0.53,1.72) | 0.59(0.08,4.10) | 1.27(0.33,4.90) | 0.927 |
| Model Ⅱ | *1（Reference）* | 0.83(0.44,1.56) | 0.55(0.08,3.88) | 1.12(0.30,4.26) | 0.873 |
| Model Ⅲ | *1（Reference）* | 0.86(0.45,1.64) | 0.58(0.08,4.11) | 1.12(0.30,4.24) | 0.917 |

Abbreviations: GDM, gestational diabetes mellitus; HDP, hypertensive disorders of pregnancy; PPH, postpartum hemorrhage; AGA, appropriate-for-gestational age; LGA, large-for-gestational age; SGA, small for gestational age; NICU, neonatal intensive care unit; T1-T4, trajectories 1-4 of serum insulin level.

Data are presented as adjusted relative risk (95% confidence interval) calculated using Poisson regression models with robust standard errors.

P-for-trend was calculated using the Poisson trend test.

Model 1 was unadjusted.

Model 2 adjusted for age at delivery, pregnancy, nulliparity, pre-pregnancy BMI, assisted reproduction.

Model 3 adjusted for factors in Model 2 in addition to higher early-pregnancy glucose levels , GDM history, and family history of diabetes.

* ^a^P < 0.001； ^b^P < 0.01；^c^P < 0.05

^d^LGA, SGA: Birth weight categories derived using gestational age-specific percentiles calculated using a 2019 Chinses reference population.

**eTable 4 Association of Gestational QUICKI Trajectories With Perinatal Complications Using the Low QUICKI Trajectory as Reference Group**

| **Perinatal complications** | **Q1** | **Q2** | **Q3** | **Q4** | ***P*-value** |
| --- | --- | --- | --- | --- | --- |
|  | Adjusted relative risk (95% CI) | | | |  |
| **GDM** |  |  |  |  |  |
| Model Ⅰ | *1（Reference）* | 0.55(0.42,0.71)^a^ | 0.42(0.19,0.92)^c^ | 0.36(0.20,0.68)^b^ | ＜0.001 |
| Model Ⅱ | *1（Reference）* | 0.59(0.45,0.77)^a^ | 0.47(0.21,0.96)^c^ | 0.40(0.22,0.75)^b^ | ＜0.001 |
| Model Ⅲ | *1（Reference）* | 0.61(0.47,0.80)^a^ | 0.51(0.23,1.17) | 0.42(0.23,0.78)^b^ | 0.001 |
| **HDP** |  |  |  |  |  |
| Model Ⅰ | *1（Reference）* | 0.29(0.18,0.47)^a^ | 0.27(0.16,0.50)^a^ | 0.17(0.04,0.59)^b^ | ＜0.001 |
| Model Ⅱ | *1（Reference）* | 0.34(0.20,0.61)^b^ | 0.29(0.15,0.57)^b^ | 0.22(0.05,0.86)^c^ | 0.001 |
| Model Ⅲ | *1（Reference）* | 0.34(0.19,0.62)^a^ | 0.30(0.14,0.58)^b^ | 0.22(0.05,0.87)^c^ | 0.001 |
| **PPH** |  |  |  |  |  |
| Model Ⅰ | *1（Reference）* | 1.16(0.76,1.75) | 0.83(0.30,2.30) | 0.87(0.41,1.87) | 0.491 |
| Model Ⅱ | *1（Reference）* | 1.25(0.81,1.93) | 0.97(0.34,2.78) | 1.10(0.47,2.19) | 0.633 |
| Model Ⅲ | *1（Reference）* | 1.18(0.75,1.85) | 0.82(0.28,2.40) | 0.94(0.43,2.05) | 0.745 |
| **LGA&**  **Macrosomia** |  |  |  |  |  |
| Model Ⅰ | *1（Reference）* | 0.61(0.38,0.96)^c^ | 0.83(0.30,2.30) | 0.33(0.10,1.06) | 0.091 |
| Model Ⅱ | *1（Reference）* | 0.72(0.44,1.17) | 1.06(0.37,2.99) | 0.42(0.13,1.41) | 0.352 |
| Model Ⅲ | *1（Reference）* | 0.68(0.41,1.12) | 0.89(0.30,2.62) | 0.39(0.12,1.30) | 0.285 |
| **SGA** |  |  |  |  |  |
| Model Ⅰ | *1（Reference）* | 1.16(0.65,2.07) | 1.16(0.35,3.92) | 0.82(0.28,2.41) | 0.711 |
| Model Ⅱ | *1（Reference）* | 0.91(0.50,1.66) | 0.82(0.24,2.74) | 0.56(0.18,1.75) | 0.790 |
| Model Ⅲ | *1（Reference）* | 0.80(0.43,1.51) | 0.60(0.16,2.21) | 0.48(0.15,1.53) | 0.627 |
| **NICU** |  |  |  |  |  |
| Model Ⅰ | *1（Reference）* | 0.98(0.60,1.59) | 0.79(0.25,2.58) | 1.39(0.67,2.85) | 0.725 |
| Model Ⅱ | *1（Reference）* | 1.10(0.65,1.87) | 0.97(0.29,3.24) | 1.69(0.79,3.61) | 0.548 |
| Model Ⅲ | *1（Reference）* | 1.06(0.62,1.82) | 0.86(0.24,3.03) | 1.60(0.74,3.46) | 0.582 |

Abbreviations: GDM, gestational diabetes mellitus; HDP, hypertensive disorders of pregnancy; PPH, postpartum hemorrhage; AGA, appropriate-for-gestational age; LGA, large-for-gestational age; SGA, small for gestational age; NICU, neonatal intensive care unit; T1-T4, trajectories 1-4 of serum insulin level.

Data are presented as adjusted relative risk (95% confidence interval) calculated using Poisson regression models with robust standard errors.

P-for-trend was calculated using the Poisson trend test.

Model 1 was unadjusted.

Model 2 adjusted for age at delivery, pregnancy, nulliparity, pre-pregnancy BMI, assisted reproduction.

Model 3 adjusted for factors in Model 2 in addition to higher early-pregnancy glucose levels , GDM history, and family history of diabetes.

* ^a^P < 0.001； ^b^P < 0.01；^c^P < 0.05

^d^LGA, SGA: Birth weight categories derived using gestational age-specific percentiles calculated using a 2019 Chinses reference population.

**eTable5 Sensitivity Analysis: Association of Posterior Probabilities of Gestational Trajectories With Perinatal Complications Using the Low Level Trajectory as Reference**

| **Perinatal complications** | Adjusted relative risk (95% confidence interval) | | | |
| --- | --- | --- | --- | --- |
|  | **F1** | **F2** | **F3** | **F4** |
| **GDM** | 1(*Reference*) | 2.05(1.29,3.24)^a^ | 3.03(1.87,4.90)^a^ | 1.66(0.56,4.97) |
| **HDP** | 1(*Reference*) | 2.38(0.78,7.27) | 5.16(1.67,15.97)^b^ | 7.87(1.72,36.07)^b^ |
| **PPH** | 1(*Reference*) | 1.44(0.82,2.53) | 0.67(0.31,1.46) | 2.22(0.78,6.30) |
| **LGA**^d^ **& Macrosomia** | 1(*Reference*) | 0.89(0.45,1.76) | 1.16(0.50,2.69) | 0.67(0.08,5.60) |
| **SGA**^d^ | 1(*Reference*) | 1.36(0.68,2.74) | 1.19(0.43,3.34) | 0.82(0.10,7.00) |
| **NICU** | 1(*Reference*) | 1.09(0.57,2.07) | 0.93(0.39,2.19) | 1.20(0.27,5.29) |
|  | **H1** | **H2** | **H3** | **H4** |
| **GDM** | 1(*Reference*) | 1,87(1.34,2.61)^a^ | 2.03(1.02,3.99)^c^ | 0.68(0.17,2.71) |
| **HDP** | 1(*Reference*) | 2.45(1.25,4.80)^b^ | 1.76(0.46,6.74) | 3.88(1.38,12.53)^b^ |
| **PPH** | 1(*Reference*) | 0.57(0.31,1.05) | 0.35(0.06,2.10) | 1.74(0.66,4.57) |
| **LGA & Macrosomia** | 1(*Reference*) | 1.34(0.69,2.60) | 2.61(1.04,6.53)^c^ | 0.80(0.12,5.38) |
| **SGA** | 1(*Reference*) | 0.45(0.19,1.08) | 0.58(0.33,1.04) | 0.77(0.12,5.45) |
| **NICU** | 1(*Reference*) | 0.93(0.49,1.76) | 0.55(0.07,4.40) | 1.27(0.33,4.90) |
|  | **Q1** | **Q2** | **Q3** | **Q4** |
| **GDM** | 1(*Reference*) | 0.53(0.37,0.74)^a^ | 0.23(0.07,0.76)^c^ | 0.40(0.20,0.82)^c^ |
| **HDP** | 1(*Reference*) | 0.36(0.17,0.74)^a^ | 0.32(0.13,0.61)^c^ | 0.20(0.05,0.79)^c^ |
| **PPH** | 1(*Reference*) | 1.24(0.74,2.08) | 0.58(0.16,2.09) | 0.71(0.28,1.81) |
| **LGA & Macrosomia** | 1(*Reference*) | 0.50(0.27,1.03) | 0.87(0.28,2.69) | 0.31(0.09,1.01) |
| **SGA** | 1(*Reference*) | 1.85(0.89,3.87) | 2.00(0.59,6.82) | 1.12(0.34,3.64) |
| **NICU** | 1(*Reference*) | 0.95(0.53,1.71) | 0.71(0.18,2.78) | 1.10(0.45,2.68) |
| Abbreviations: GDM, gestational diabetes mellitus; HDP, hypertensive disorders of pregnancy; PPH, postpartum hemorrhage; LGA, large-for-gestational age; SGA, small for gestational age; NICU, neonatal intensive care unit. Data are presented as adjusted relative risk (95% confidence interval) calculated using Poisson regression models with robust standard errors.  *P*-for-trend was calculated using the Poisson trend test.  Model adjusted for age at delivery, pregnancy, nulliparity, pre-pregnancy BMI, assisted reproduction, higher early-pregnancy glucose levels , GDM history, and family history of diabetes. ^a^P < 0.001; ^b^P < 0.01; ^c^P < 0.05 ^d^LGA, SGA: Birth weight categories derived using gestational age-specific percentiles calculated using a 2019 Chinses reference population. | | | | |

**eTable6 Sensitivity Analysis: Association of Summary Insulin Resistance Metrics with Perinatal Complications Using Logistic Regression**

| **Summary Metrics** | | **GDM** | | **HDP** | |
| --- | --- | --- | --- | --- | --- |
|  |  | **OR(95%CI)** | **P-value** | **OR(95%CI)** | **P-value** |
| Mean | FINS | 1.21 (1.02,1.44) | 0.031 | 1.54 (1.24,1.91) | ＜0.001 |
|  | HOMA-IR | 1.36 (1.19,1.56) | ＜0.001 | 1.70 (1.42,2.02) | ＜0.001 |
|  | QUICKI | 0.98 (0.97,0.99) | ＜0.001 | 0.97 (0.95,0.98) | ＜0.001 |
| Max | FINS | 2.57 (1.41,4.72) | 0.002 | 10.87 (4.62,25.57) | ＜0.001 |
|  | HOMA-IR | 1.08 (1.01,1.14) | 0.020 | 1.18 (1.10,1.27) | ＜0.001 |
|  | QUICKI | 0.98 (0.98,0.99) | ＜0.001 | 0.98 (0.97,0.99) | ＜0.001 |
| Last | FINS | 1.34 (0.76,2.35) | 0.305 | 4.19 (1.80,9.75) | 0.001 |
|  | HOMA-IR | 1.04 (0.97,1.11) | 0.291 | 1.13 (1.04,1.22) | 0.003 |
|  | QUICKI | 0.48 (0.21,1.75) | 0.180 | 0.45 (0.20,0.76) | 0.019 |
| Cumulative | FINS | 2.00 (1.53,2.62) | ＜0.001 | 4.31 (2.73,6.79) | ＜0.001 |
|  | HOMA-IR | 1.11 (1.06,1.16) | ＜0.001 | 1.19 (1.13,1.27) | ＜0.001 |
|  | QUICKI | 0.94 (0.92,0.96) | ＜0.001 | 0.89 (0.86, 0.93) | ＜0.001 |
| Standard Deviation | FINS | 0.18 (0.03,0.94) | 0.042 | 2.26 (0.24,21.55) | 0.478 |
|  | HOMA-IR | 1.05 (0.92,1.19) | 0.50 | 1.26 (1.09,1.45) | 0.001 |
|  | QUICKI | 0.93 (0.84,1.04) | 0.233 | 1.01 (0.85,1.21) | 0.876 |
| Time-weighted Average | FINS | 4.70 (2.23,9.94) | ＜0.001 | 32.79 (9.53,112.77) | ＜0.001 |
|  | HOMA-IR | 1.20 (1.08,1.33) | 0.001 | 1.39 (1.21,1.58) | ＜0.001 |
|  | QUICKI | 0.87 (0.82,0.92) | ＜0.001 | 0.77 (0.70,0.85) | ＜0.001 |
| Abbreviations: GDM, gestational diabetes mellitus; HDP, hypertensive disorders of pregnancy;  FINS, fasting serum insulin; HOMA-IR, homeostasis model assessment of insulin resistance; QUICKI, quantitative insulin sensitivity check index. Data are presented as odds ratio (95% confidence interval) calculated using Logistic Regression. | | | | | |

**eTable 7 Associations of Individual-Level Multidomain Factors With Each Serum Insulin Level Trajectory**

| Individual-level multidomain factors | F2 | F3 | F4 |
| --- | --- | --- | --- |
|  | Adjusted relative risk (95% confidence interval)* | | |
| Age at delivery |  |  |  |
| 18-24 | 1(Reference) | 1(Reference) | 1(Reference) |
| 25-29 | 0.97 (0.95 ,1.00 ) | 0.98 (0.93 ,1.02 ) | 1.03 (1.01 ,1.05 )^a^ |
| 30-34 | 0.98 (0.97 ,1.00 ) | 0.99 (0.96 ,1.02 ) | 1.12 (1.11 ,1.14 )^a^ |
| 35-55 | 0.97 (0.94 ,1.01 ) | 0.98 (0.93 ,1.02 ) | 1.04 (1.01 ,1.06 )^a^ |
| BMI(kg/m2)b |  |  |  |
| Underweight | 0.89 (0.86 ,0.93 )^a^ | 0.81 (0.78 ,0.84 )^a^ | 1.01 (0.88 ,1.16 ) |
| Normal | 1(Reference) | 1(Reference) | 1(Reference) |
| Overweight | 1.05 (1.03 ,1.08 )^a^ | 1.29 (1.22 ,1.35 )^a^ | 1.15 (1.02 ,1.32 )^a^ |
| Obsese | 1.01 (0.99 ,1.01) | 1.05 (1.02 ,1.10)^a^ | 1.00 (0.99 ,1.00 ) |
| Race |  |  |  |
| Han | 1(Reference) | 1(Reference) | 1(Reference) |
| Ethnic minorities | 1.00(0.98,1.02) | 1.01(0.98,1.04) | 1.00(0.94,1.07) |
| Residential area |  |  |  |
| Guangdong | 1(Reference) | 1(Reference) | 1(Reference) |
| The other provinces | 0.99(0.95,1.03) | 0.97(0.91,1.03) | 1.02(0.90,1.16) |
| Assisted reproduction |  |  |  |
| No | 1(Reference) | 1(Reference) | 1(Reference) |
| Yes | 1.03(0.98,1.07) | 1.01(0.96,1.06) | 1.06(0.95,1.19) |
| Nulliparous |  |  |  |
| No | 1(Reference) | 1(Reference) | 1(Reference) |
| Yes | 0.99(0.91,1.07) | 1.00(0.89,1.13) | 0.89(0.67,1.17) |
| Family history of diabetesc |  |  |  |
| No | 1(Reference) | 1(Reference) | 1(Reference) |
| Yes | 1.35(0.89,2.05) | 2.00(1.19,3.37)^a^ | 2.53(1.09,5.87)^a^ |
| Higher early-pregnancy glucose levelsd |  |  |  |
| No | 1(Reference) | 1(Reference) | 1(Reference) |
| Yes | 1.12(1.07,1.17)^a^ | 1.32(1.14,1.52)^a^ | 1.06(0.83,1.36) |
| Abbreviations: BMI, body mass index(calculated by pre-pregnancy weight in kilograms divided by height in meters squared); F2, middle insulin level trajectory; F3, high insulin level trajectory; F4, special insulin trajectory. *Data are presented as adjusted relative risk (95% confidence interval) calculated using Poisson regression models with robust standard errors with T1 low insulin level trajectory as the reference group for the given trajectory outcome, adjusted fora ge at delivery, race and residential area, multiparity, pre-pregnancy body mass index, history of GDM, history of macrosomia, a immediate family history of diabetes, higher fasting glucose levels, HbA1c ≥6% in early pregnancy. ^a^ P < 0.05, corrected by the Benjamini-Hochberg method ^b^ Pre-pregnancy BMI was categorized as follows: underweight (<18.5 kg/m2), normal (18.5-24.9 kg/m2), overweight (25.0-29.9 kg/m2), obese (≥30.0 kg/m2).  ^c^ Family history of diabetes was limited to first-degree relatives who are related by blood. ^d^ Higher fasting glucose levels: ≥ 5.1 mmol/L in early pregnancy. | | | |
